# Disseminated tuberculosis associated with reactive arthritis of Poncet in an immunocompetent patient^☆^^☆☆^

**DOI:** 10.1016/j.abd.2019.08.031

**Published:** 2020-03-20

**Authors:** Juliana de Oliveira Alves Calado, Anna Carolina Miola, Maria Regina Cavariani Silvares, Silvio Alencar Marques

**Affiliations:** Department of Dermatology and Radiotherapy, Faculdade de Medicina de Botucatu, Universidade Estadual Paulista, Botucatu, SP, Brazil

**Keywords:** Arthritis, reactive, Dermatology, Tuberculosis, cutaneous

## Abstract

Cutaneous tuberculosis is a rare extrapulmonary manifestation of tuberculosis which, like disseminated tuberculosis, commonly occurs in immunocompromised patients. Poncet reactive arthritis is a seronegative arthritis affecting patients with extrapulmonary tuberculosis, which is uncommon even in endemic countries. We report a previously healthy 23-year-old male patient with watery diarrhea associated with erythematous ulcers on the lower limbs and oligoarthritis of the hands. Histopathological examination of the skin showed epithelioid granulomatous process with palisade granulomas and central caseous necrosis. AFB screening by Ziehl–Neelsen staining showed intact bacilli, the culture was positive for *Mycobacterium tuberculosis*, and colonoscopy revealed multiple shallow ulcers. Disseminated tuberculosis associated with reactive Poncet arthritis was diagnosed, with an improvement of the clinical and skin condition after appropriate treatment.

## Introduction

Although tuberculosis (TB) is one of the most common diseases in humans, its cutaneous form is rare and represents about 1–2% of cases of extrapulmonary TB, which corresponds to 10% of the total cases.^1^, ^2^ Clinically, there are three types: endogenous cutaneous TB (by hematogenous spread), exogenous cutaneous TB (by inoculation) or tuberculids (hypersensitivity reaction to *Mycobacterium tuberculosis*)^3^ The treatment usually performed with rifampicin, isoniazid, pyrazinamide and ethambutol (RIPE) provides resolution of cutaneous TB cases.

Tuberculosis is considered multifocal when there is involvement of at least two extrapulmonary sites, with or without pulmonary involvement. It accounts for one-third of the mortality among patients infected with the human immunodeficiency virus (HIV), but it can also affect immunocompetent patients.^4^

Poncet's reactive arthritis was described by Antonin Poncet in 1897 as a TB-associated polyarthritis and is currently defined by polyarthritis or oligoarthritis in the presence of TB, usually visceral. Despite the involvement of the joints, no bacilli are found in the joint fluid of the symptomatic patients,^5^, ^6^ and the patients present an improvement of the joint condition after adequate TB treatment.^7^ With this case, we report the rare association of cutaneous TB with reactive arthritis of Poncet, as well as general improvement of the clinical picture after adequate treatment with RIPE scheme.

## Case report

**Figure 1:**
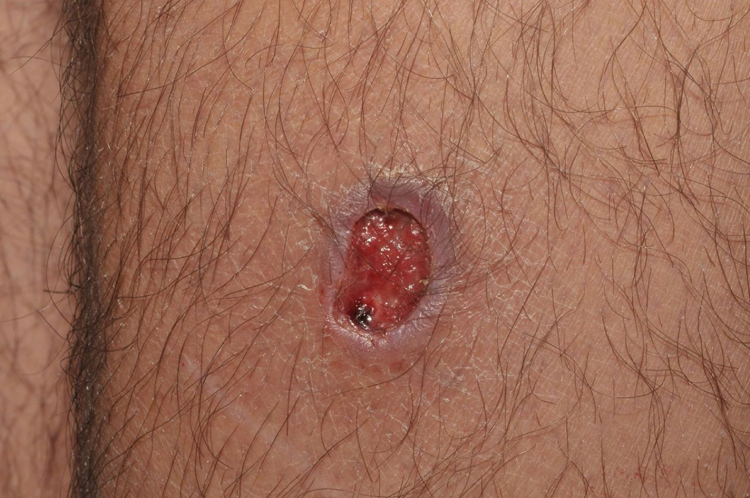
Well-delimited ulcer with clean floor on the leg.

**Figure 2:**
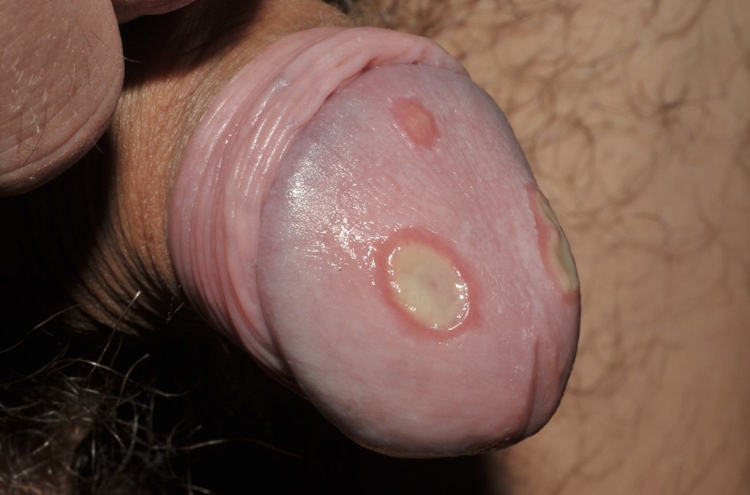
Shallow ulcers on the penis, with fibrin-containing floor.

**Figure 3:**
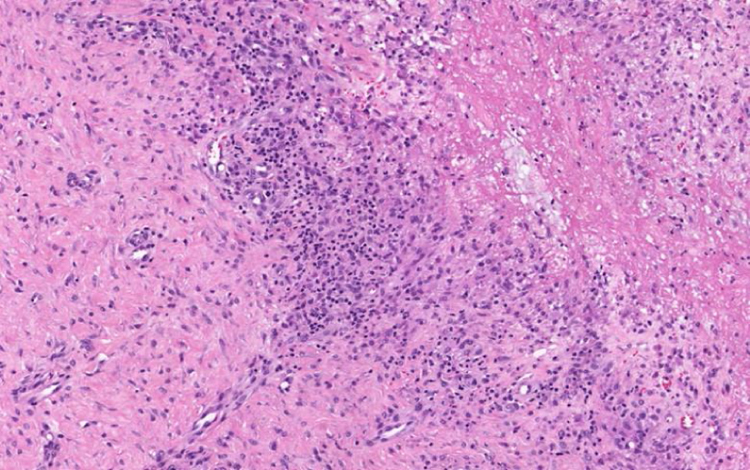
Epithelioid granulomatous process in the deep dermis.

**Figure 4:**
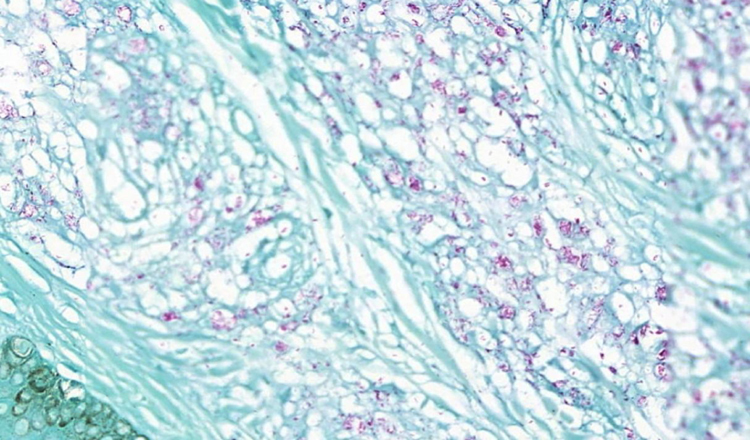
Presence of multiple solid bacilli (Ziehl–Neelsen, ×40).

**Figure 5:**
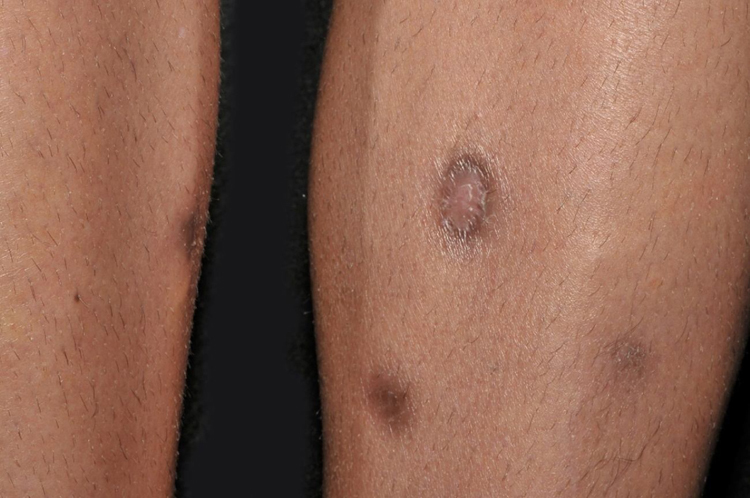
Complete healing of leg lesions after two months with RIPE treatment.

**Figure 6:**
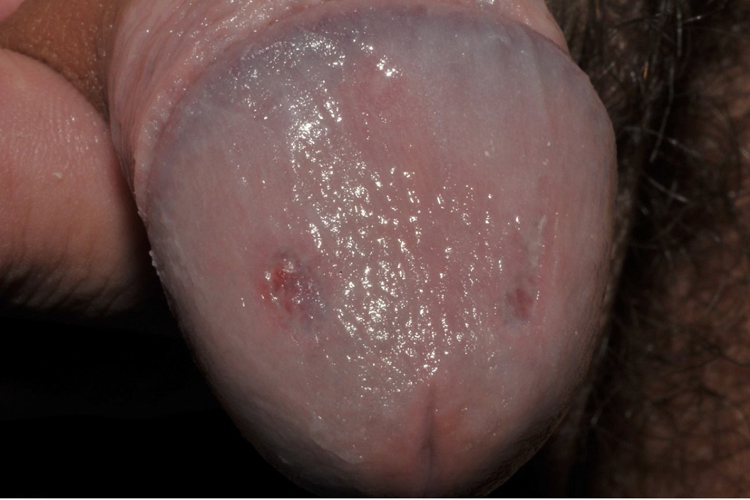
Complete healing of penile lesions after two months with RIPE treatment.

A 23-year-old male patient, previously healthy, started with watery diarrhea, abdominal pain and weight loss of 6 kg, associated with nocturnal episodes of fever, 9 months before admission. He denied the consumption of alcohol or illicit drugs. The patient evolved with multiple ulcers with necrotic crust, some with granular floor and raised borders, associated with soft erythematous painful nodules on the lower limbs and ulcers of the fibrinous floor in the glans (Figure 1, Figure 2). In addition, he complained of arthralgia in the phalanges, with signs of oligoarthritis of small joints on physical examination. He showed normal exam of the joint fluid and laboratory tests, except for the presence of leukocytes in the feces, and had no previous history of any kind of immunosuppression. At colonoscopy, multiple shallow ulcers covered by thick fibrin associated with enanthem, mainly in the sigmoid, and aftoid ulcers in the proximal rectum were evidenced. The tuberculin sensitivity test (PPD) was negative and the chest X-ray had no alterations. Anatomopathological examination of one of the ulcers was performed on the lower limb, which revealed an epithelioid granulomatous process with palisade granulomas and central caseous necrosis. The study of acid-fast bacilli (AFB) by Ziehl-Neelsen staining showed intact bacilli (Figure 3, Figure 4), and culture was positive for *M. tuberculosis*, confirming the diagnostic hypothesis of cutaneous TB. The histopathological analysis of the intestinal biopsy revealed a mild inflammatory infiltrate without the presence of bacilli. After the beginning of the RIPE treatment regimen, the patient evolved with complete healing of the ulcers (Figure 5, Figure 6) and gradual resolution of the diarrhea and oligoarthritis.

## Discussion

Tuberculosis is an important public health problem, especially in underdeveloped countries. In the last decades, it has been reclassified as a re-emerging disease due to increased poverty, malnutrition, increased coinfection with HIV, use of immunosuppressive drugs and cases resistant to the drugs used in treatment.

Multifocal forms are rare and account for 9–10% of cases of extrapulmonary disease. It accounts for one-third of the mortality among HIV-infected patients, but may also affect immunocompetent individuals, with mortality rates ranging from 16% to 25%, although the risk of developing extrapulmonary lesions is proportional to the degree of immunodeficiency.^8^ Unlike miliary TB, it may not affect the lungs, as in the case herein reported.^9^

There are hypotheses trying to justify the occurrence of multifocal TB in immunocompetent patients, such as the intensity of transmission in the community, Mendelian susceptibility syndrome to mycobacterial infections due to the existence of defects of interleukin-12, and malnutrition, among others.^4^, ^8^ In the case reported, there was no personal or family history of immunosuppression; however, at the first visit, the patient was evidently malnourished, which may justify the presence of multiple sites of TB.

The diagnosis of cutaneous TB should be differentiated from leishmaniasis, leprosy, cat scratch disease and deep fungal infections. The concomitant presence of pulmonary TB or in other organs increases the diagnostic probability, which can be confirmed by PPD, microbiological examination with culture and/or PCR.^8^ In the case reported here, through the positive culture and the presence of numerous AFB in Ziehl–Neelsen staining examination, the diagnosis was confirmed and the patient was submitted to RIPE scheme appropriately, with improvement of the condition at the end of treatment.

Intestinal TB may have a variable and nonspecific clinical picture, and anatomopathological examination may not indicate AFB infection in up to 29% of cases. However, colonoscopy may reveal shallow ulcers in the ileocecal region, with improvement after treatment, as occurred with the patient reported.^10^

The description of Poncet's reactive arthritis consists in polyarthritis or oligoarthritis associated with active TB, without the presence of bacilli in the joints.^6^ Its pathogenesis is poorly understood, but appears to be immune-mediated. The duration of symptoms may vary from months to years, and cases of oligoarthritis predominate, with improvement after use of non-steroidal anti-inflammatory drugs, usually after 5 months of onset of symptoms. The diagnosis is mostly clinical and there are no well defined diagnostic criteria, given the low frequency of the condition. Most patients improve during or after TB treatment, and cases of chronification are rare. Its clinical diagnosis is important, since clinical management should be performed together with an experienced rheumatologist, in view of the consequences of inadequate immunosuppression in a patient infected with *M. tuberculosis*. In this case, the rheumatology team chose hydroxychloroquine, avoiding prolonged immunosuppression, with good control of the disease during RIPE scheme and resolution of oligoarthritis at the end of treatment. This case alerts us to the possibility of severe, multifocal, cutaneous expression, requiring special care and multidisciplinary care, making it necessary to be recognized by the dermatologist in countries where TB remains endemic.

## Financial support

None declared.

## Authors’ contributions

Juliana Alves Calado: Approval of the final version of the manuscript; elaboration and writing of the manuscript; critical review of the literature; critical review of the manuscript.

Anna Carolina Miola: Approval of the final version of the manuscript; elaboration and writing of the manuscript; critical review of the literature; critical review of the manuscript.

Maria Regina Cavariani Silvares: Approval of the final version of the manuscript; critical review of the literature; critical review of the manuscript.

Silvio Alencar Marques: Approval of the final version of the manuscript; critical review of the literature; critical review of the manuscript.

## Conflicts of interest

None declared.
